# Comparative evaluation of rapid diagnostic test and PCR-based diagnostic assay for identification of trypanosomes in cattle of Apac and Kiryandongo districts, Uganda: A cross sectional study

**DOI:** 10.1186/s12917-024-04436-7

**Published:** 2024-12-19

**Authors:** Daniel Kizza, Rose Azuba, Eddie Wampande, Rodney Okwasiimire, Lillian Owembabazi, Wangoola Mandela, Charles Waiswa, Agricola Odoi

**Affiliations:** 1 Department of Livestock and Industrial Resources, College of Veterinary Medicine Animal Resources and Biosecurity, Makerere University, Kampala, Uganda; 2 Department of Veterinary Pharmacy, Clinical, and Comparative Medicine, College of Veterinary Medicine Animal Resources and Biosecurity, Makerere University, Kampala, Uganda; 3 Department of Veterinary Pharmacy, Clinical, and Comparative Medicine, College of Veterinary Medicine Animal Resources and Biosecurity, Central Diagnostic Laboratory, Makerere University, Kampala, Uganda; 4 Coordinating Office for the Control of Trypanosomiasis in Uganda (COCTU), Kampala, Uganda; 5 College of Veterinary Medicine, Biomedical and Diagnostic Sciences, University of Tennessee, Knoxville, US; 6 Department of Agricultural and Environmental Sciences, School of Agricultural and Applied Sciences, Bugema University, Luweero, Uganda; 7 College of Veterinary Medicine Animal Resources and Biosecurity, Central Diagnostic Laboratory, Makerere University, Kampala, Uganda

**Keywords:** Rapid diagnostic test, mini PCR, Performance, Trypanosomes, Cattle, Apac. Kiryandongo, Uganda

## Abstract

**Background:**

Diagnostic tests and knowledge of their diagnostic accuracies are important for animal trypanosomosis surveillance and treatment.

**Methodology:**

: A cross-sectional study was conducted in November 2021 to compare the performance of rapid diagnostic test (RDT) and PCR-based assay for the detection of trypanosome infections. Random sampling and probability proportional to size sampling were used to sample study households and animals from the sampled household respectively. Animals were screened for the presence of trypanosomes using both tests. A total of 100 cattle (52 from Apac and 48 from Kiryandongo districts) were included in the study. The percentage of positive tests, sensitivity, and specificity of the tests using mini PCR as a reference were computed. Cohen’s kappa statistics was computed to assess how well the rapid diagnostic test agrees with the mini PCR. McNemar’s statistic assessed if the proportion of positive tests identified by mini PCR significantly differed from that identified by the rapid diagnostic test.

**Results:**

The mini PCR test detected 31.2% *Trypanosome spp* positive samples in Kiryandongo while it detected only 5.7% positive samples in Apac district. The rapid diagnostic test (RDT) analysis detected 91.6% *Trypanosome spp* positive samples in Kiryandongo district and only 46.1% in Apac district. *T. congolense* was the most common *Trypanosoma* species identified in Apac (36.5%) and Kiryandongo (47.9%) by the RDT. The percentage of each of the *Trypanosome* species (*T.vivax*, *T.congolense*, and mixed infection *of T.vivax* and *T. congolense*) detected by RDT differed significantly (*p* < 0.001) between the two districts. The RDT had a high sensitivity of 94.4% (95% Confidence Interval (CI): 72.7–99.9) but a very low specificity of 36.6% (95% CI: 26.2–48.0). The kappa test showed evidence of only a slight agreement (kappa = 0.1547, Accuracy = 0.48 (95% Confidence Interval (CI): 0.379,0.5822) between the two tests. The observed agreement between the tests was 47% while the expected agreement was 37.84%.

**Conclusion:**

This study found high sensitivity but low specificity of RDT using mini PCR as a reference.

**Supplementary Information:**

The online version contains supplementary material available at 10.1186/s12917-024-04436-7.

## Background

African trypanosomes cause Animal African Trypanosomosis (AAT) and Human African Trypanosomosis (HAT) which are debilitating diseases [1]. Most trypanosomes are transmitted by tsetse flies (*Glossinidae*) which are approximately found in more than 70% of Uganda [2]. The main trypanosome species of economic importance that cause trypanosomosis in cattle are *T. brucei brucei*, *T. vivax* and *T. congolense* [3]. Both AAT and HAT have impeded the development of the African continent and are major constraints to mixed crop-livestock agriculture, food security and human health [4]. The economic losses due to AAT are estimated at US $ 4.75 billion per year [5] and more than 50 million people are at a risk of contracting HAT [6, 7].

Most livestock farmers use bait technology (insecticide treated cattle) and trypanocides to control tsetse fly population and the disease, respectively. Diagnosis of trypanosomosis in Uganda is based mainly on clinical signs. Disease control is limited by parasite resistance and high toxicity of tsetse chemical control [1]. In Uganda, animal disease diagnostic services at the districts are not well developed to provide quality services to the farmers and field veterinarians. The implication is that farmers and veterinarians often depend on tentative diagnosis based on clinical signs since no laboratory diagnostic services are readily available. This inevitably leads to incorrect diagnoses resulting in wrong treatment and financial loss. Moreover, the development of drug resistant trypanosome strains is a serious challenge in trypanosomosis management initiatives especially in the resource constrained regions of Africa including Uganda [8], where veterinary laboratory diagnostic services are few and rarely used [9]. In Uganda, the national animal disease diagnostic laboratory (NADDEC) is located at Entebbe and the second animal disease laboratory facility is located at the College of Veterinary Medicine, Makerere University, Kampala. Both diagnostic facilities are not readily accessible from most districts in Uganda due to distance. Therefore, use of rapid diagnostic tests (RDT) and field based portable mini PCR machines provide practical alternatives to these scarce and inaccessible laboratory facilities. Moreover, these rapid and portable diagnostic kits are much cheaper, use less sophisticated equipment and do not need a lot of training and expertise to use.

Kiryandongo district in Bunyoro sub-region and Apac district in Lango sub-region are some of the districts with the highest numbers of cattle in Uganda [10], but have no functioning livestock disease diagnostic laboratories [10]. The diagnostic approaches and successful treatment of animal trypanosomosis depends on timely and accurate diagnosis of the disease. Although Polymerase Chain Reaction (PCR) has been widely used for the detection of trypanosomes and has relatively good sensitivity (65–88%) and almost perfect specificity (99–100%) [11] in laboratory settings [12, 13], it is not readily available in resource poor and rural settings of Uganda. Unfortunately, the application of Rapid Diagnostic Tests (RDTs) is still very limited in Uganda and yet they provide excellent alternatives in these resource poor settings. Therefore, the objective of this study was to compare the performance of rapid diagnostic tests and mini PCR based assays for the detection of trypanosome infections in two cattle corridor districts of Uganda.

## Methods

### Study design and area

#### Study area

**Fig. 1:**
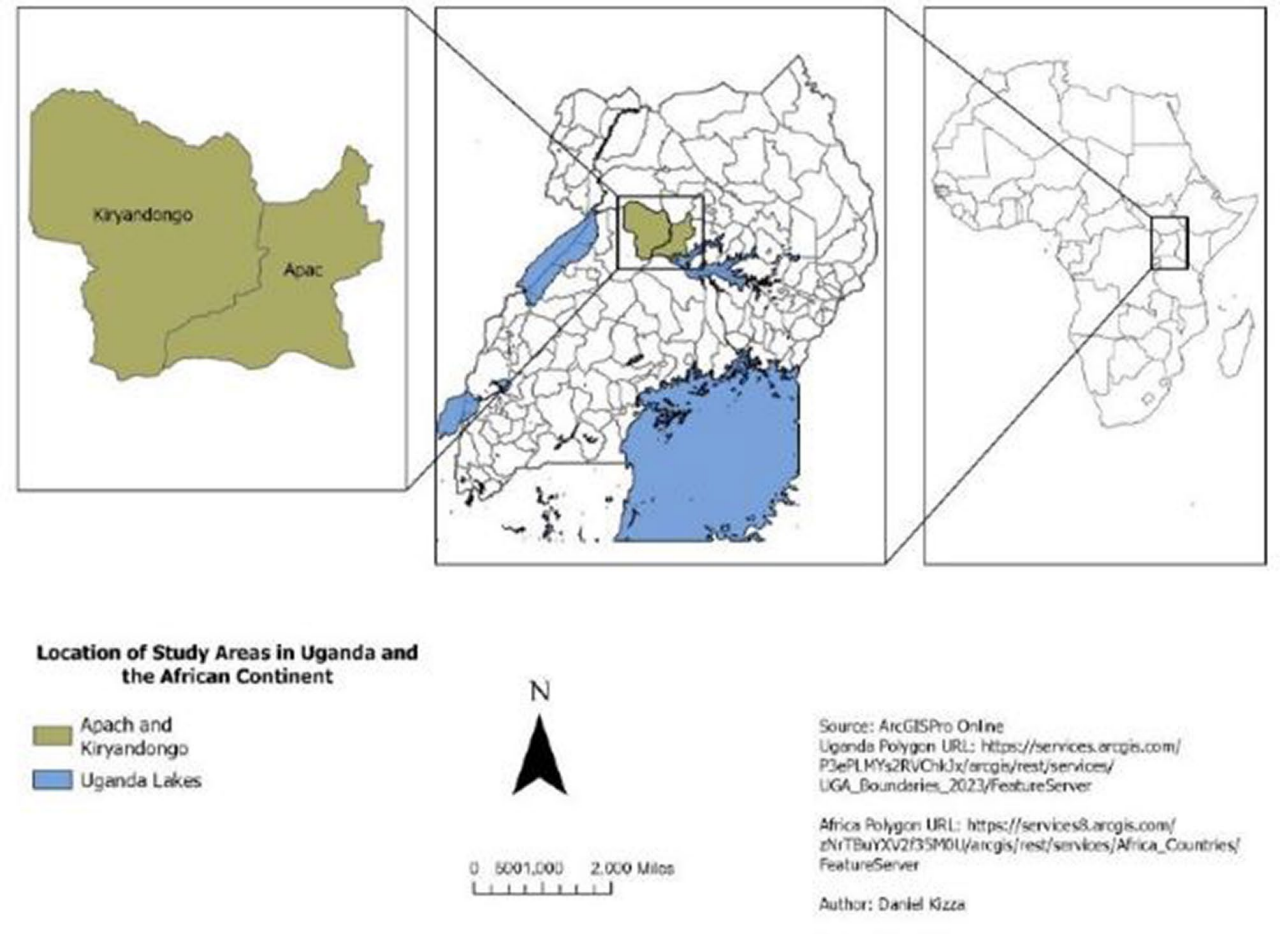
Map of Uganda showing the study districts

This study was conducted in Apac town council and Ibuje sub-counties of Apac district and Kiryandongo sub-county of Kiryandongo district (Fig. 1). These areas were selected due to their proximity to Murchision Falls National Park, an area that has a high risk of cattle trypanosomosis [3]. Apac district is located in Northern Uganda with a topography characterized by low plains and rolling hills with an average altitude of 1,150 m above sea level. It has mainly dry Savannah vegetation with reddish brown clay loam soils which are favorable for agriculture. The district has both dry and wet seasons with the wet season running from April to November reporting annual rainfall of 1,330 mm. Most of the population in this area is involved in subsistence agriculture [14]. Kiryandongo district is located in mid-western Uganda bordering Nwoya district in the north, Apac in the east and Masindi in the south west. The topography is mostly plateau with average altitude of 1,295 m above sea level and the main type of soils are sandy loam and clay loam. The district has a bimodal rainfall pattern with the wet season occurring in August to November. The area receives an average annual rainfall of 1,200 mm [15]. The majority of the population is involved in Agriculture (food crops, livestock and aquaculture) with cattle being the major source of income.

#### Study design

This was a cross-sectional study and data collection was conducted in November 2021. A sampling frame of all cattle keeping households was obtained from the sub-county veterinary office. A simple random sampling strategy was then used to obtain study households from the sampling frame. Probability proportional to size sampling (PPS) strategy was used to sample animals from sampled households. PPS is a method of sampling from a finite population in which a size measure is available for each population unit before sampling and where the probability of selecting a unit is proportional to its size [16].

#### Eligibility criteria

Cattle keeping households that consented to participated in the study.

#### Variables

Blood drawn from study animals was tested for presence of *trypanosomes* spp using both the rapid diagnostic test and mini PCR.

## Data sources/measurements

### Blood sample collection

Blood samples were obtained from marginal ear veins and collected into two micro-hematocrit tubes. One of the collected blood samples in a micro-hematocrit tube was applied onto the designated sample area of a classic Whatman^®^ Flinders Technology Associates (FTA^®^) card. The blood samples in the second micro-hematocrit tube was kept for the rapid diagnostic test.The sample cards were then labeled with a unique field number for each sampled animal and placed on a solid surface for air drying prior to processing for analysis on the miniPCR^®^.

## Laboratory analysis of samples

### Rapid diagnostic testing

Detection of antibodies against *T.vivax* and *T. congolese* in whole blood samples was done using the Ceva VerYDiag rapid diagnostic kit according to the manufacturer’s instructions [17]. Briefly, this was done by adding one drop (approximately 20 µl) of blood sample in a microhematocrit tube to the designated sample area on the test strip cassette. This was followed by adding one drop of the dilution buffer to the sample area and waiting for approximately 10 min for the test combo to react prior to reading results. No results were read later than 15 min post mixing of blood and buffer.

The test assay was considered valid following formation of a red line in the control region of the result window on the test combo after 10 min. Presence of a red line at the *Tc* and/or *Tv* regions of the result window indicated presence of antibodies against *T.congolense* and/or *T.vivax* in the sample.

### DNA extraction

To obtain DNA from the FTA^®^ sample cards, four discs were punched out of a given sample area with a 3.0-mm Harris Micro Punch into a labelled microfuge tube. The Harris Micro Punch was cleaned between samples by spraying with 70% ethanol and punching a minimum of five times from a clean Whatman^®^ filter paper. Four of these discs were randomly selected and processed alongside the test samples as the negative extraction control. The sample discs were then cleaned by incubating with 1,000 µl of FTA^®^ purification reagent for 20 min at room temperature. This was followed by a 15 min incubation at room temperature with 1,000 µl of Tris EDTA (TE) buffer. The incubation with the FTA^®^ purification reagent was carried out twice while incubation with TE buffer was carried out three times to ensure maximum removal of the purification reagent from the discs. The clean sample discs were then carefully transferred into a fresh correspondingly labelled 1.5mL microfuge tube and incubated at 37 ºC for 45 min to dry out the TE buffer. An elution solution of 5% (w/v) Chelex 100 resin was then added (100 µl) to the dried sample discs and heated at 90ºC for 30 min on a heat block. The tubes were then centrifuged and the eluate transferred to a fresh 1.5 ml microfuge tube as ready template for PCR analysis [18–20].

### PCR Analysis

Primers targeting the internal transcribed spacer 1 (ITS1) of various trypanosome species were used in this study (ITS 1 CF, 5′-CCGGAAGTTCACCGATATTG-3′ and ITS1 BR, 5′-TTGCTGCGTTCTTCAACGAA-3′) [21, 22]. Test assays were carried out in 25 µl reaction volumes that contained 0.5 µM of each ITS1 CF and BR primers (Biolegio B.V, The Netherlands), 5 µl of EZ-PCR Load Ready™ Master Mix, 5 µl of Nuclease free water (Promega Corporation, Madison, USA) and 5 µl of the extracted DNA. PCR amplification was carried out on a mini16 thermocycler (miniPCR^®^) (Mini pcr bio™, UK) with the following parameters on the miniPCR^®^ android application: initial step at 94 ºC for 5 min, followed by 35 cycles of 94 ºC for 40 s, 58 ºC for 40 s, 72 ºC for 90 s, with a final extension at 72 ºC for 5 min [3]. Electrophoresis of the resultant amplification products plus a 50 bp standard molecular ladder (Thermo Scientific™, Waltham, Massachusetts, USA) was done in a blueGel™ Integrated electrophoresis and visualization system with 1X TBE buffer on a 1.5% agarose gel stained with GelGreen^®^ Nucleic Acid Gel Stain for 20 min.

### Sample size

The sample size was estimated using the following formula:

$$\:n=\frac{\left(Z\right)^{2}pq}{e^{2}}$$

Where n = sample size, Z = Z value 1.96 at 95% confidence level, e = desired level of precision (5%), p = expected prevalence of trypanosomosis in cattle (14.28%) [23, 24], and q = 1-p. A total of 100 animals (48 from Kiryandongo and 52 from Apac districts) were randomly sampled for inclusion in the study.

## Statistical methods

### Statistical analysis

Data were entered into Microsoft Excel^®^ 2020 spreadsheet and exported to statistical package for social scientist software (SPSS) IBM version 20 which was used to compute percentage of positive tests, sensitivity, and specificity of the tests using mini PCR as a reference. Cohen’s kappa statistics was computed to assess how well the rapid diagnostic test agrees with the mini PCR. McNemar’s statistic assessed if the proportion of positive tests identified by mini PCR significantly differed from that identified by the rapid diagnostic test [25].

## Results

### Animal characteristics

A total of 100 cattle (52 from Apac and 48 from Kiryandongo districts) were included in the study. The percentage of cattle by age and sex did not differ significantly (*P* > 0.05) between the two districts (Table 1). However, there was a significant difference (*P* < 0.05) in distribution of cattle breeds with Kiryandongo farmers rearing only local breeds while animals kept by Apac farmers were 86.5% local breeds and the rest were crossbreeds (Table 1).

**Table 1:** Comparison of distribution of cattle sampled, test results, and Trypanosoma species between Apac and Kiryandongo districts, Uganda

		District		*p*-values
Variables		Apac (*n* = 52)	Kiryandongo (*n* = 48)	
Age	Adult	61.5	77	0.071
	Heifer/steers	38.4	22.9	
Sex	Males	40.3	54.1	0.119
	Females	59.6	45.8	
Breed	Local breed	86.5	100	0.008*
	Cross breed	13.4	0	
^b^PCR results + ve		5.7	31.2	0.001*
^c^RDT results + ve		46.1	91.6	< 0.001*
Trypanosome species detected by RDT	*T. vivax (T.v)*	7.6	2	< 0.001*
	*T. Congolense (T.c)*	36.5	47.9	< 0.001*
	*T.v/T.c*	1.92	41.6	< 0.001*
Trypanosome species detected by PCR	*T. vivax (T.v)*	1.92	27	< 0.001*
	*T. Congolense (T.c)*	3.84	4.16	0.660

^**a**^p**-**values compare percentages between Apac and Kiryandongo

^b^PCR: Polymerase chain reaction

^c^RDT: Rapid diagnostic test

### Trypanosomiasis test results

**Fig. 2:**
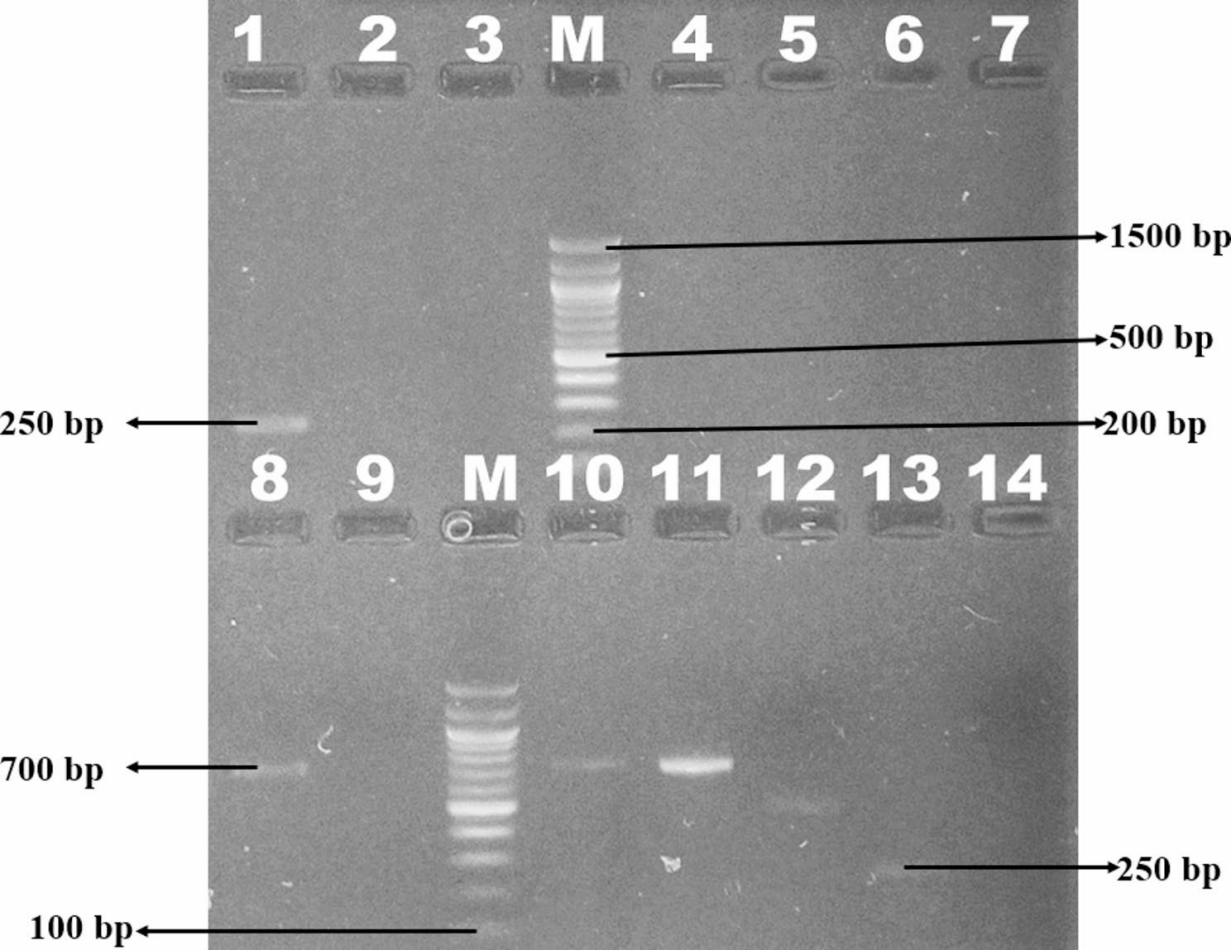
Representative gel image of the test results amplified on the miniPCR^®^ after electrophoresis on the blueGel™ Integrated electrophoresis system. Image was captured on a synegene UV transilluminator to enhance quality for publication purposes. *Lanes*: *1* 250 *bp (T. vivax)*, *M*: 100 bp DNA ladder, 4: Negative control, 8: 700 bp *(T. Congolese spp.)*, 12: 480 bp *(T. Brucei sl)*

The result from mini PCR is shown by a representative gel image in Fig. 2. Lanes 1 with 250 base pairs represents *T. vivax*; lane M with 100 base pairs represents DNA ladder; lane 4 represents negative control; lane 8 with 700 base pairs represents *T. Congolese spp.* while lane 12 with 480 base pairs represents *T. Brucei sensu latu (sl).* There was a significant difference in the proportion of test positive samples based on a comparison of the mini PCR and RDT test results between the two districts. The mini PCR test detected 31.2% *trypanosome spp* positive samples in Kiryandongo while it detected only 5.7% positive samples in Apac district (Table 1). As shown in Table 1, the RDT analyses detected 91.6% *trypanosome spp* positive samples in Kiryandongo district and only 46.1% in Apac district. Overall, T. congolense was the most common species of Trypanosoma identified in both Apac (36.5%) and Kiryandongo (47.9%). The percentage of each of the trypanosome species (*T.vivax*, *T.congolense*, and mixed infection *of T.vivax* and *T. congolense*) detected by RDT differed significantly (*p* < 0.001) between the two districts (Table 1). In Kiryandongo, RDT detected 2% as *T. vivax*, 47.9% as *T.congolense*, and 41.6% as mixed infection of *T.congolense* and *T. vivax.* (Table 1). In Apac, the RDT detected 7.6% as *T. vivax*, 36.5% as *T.congolense*, and 1.92% as mixed infections of *T.congolense* and *T. vivax* (Table 1). However, there was no significant difference (*p* = 0.660) in the percentage of *T.congolense* detected by mini PCR in the two districts (Table 1).

There was a significant difference (*p* < 0.001) in the proportions of positive samples identified by the mini PCR (17/18, 94%) and RDT (17/69, 24%) (Table 2). Moreover, the kappa test showed evidence of only a slight agreement (kappa = 0.1547, accuracy = 0.48 (95% Confidence Interval (CI): 0.379,0.5822)) between the two tests (Table 3). The observed agreement between the tests was 47% while the expected agreement was 37.84% (Table 3).

**Table 2:** Results of McNemar’s test used to compare the proportions of positive tests of RDT against mini PCR in Apac and Kiryandongo districts, Uganda

	RDT^a^		Total
**Mini PCR** ^**b**^	Test Positive	Test Negative	
Test Positive	17	1	18
Test Negative	52	30	82
Total	69	31	100

McNemar’s Test *P* < 0.001

^a^RDT= Rapid diagnostic test

^b^PCR= Polymerase chain reaction

**Table 3:** Results of kappa test evaluating the level of agreement between the rapid diagnostic tests and mini PCR in detection of Trypanosomes in Apac and Kiryandongo districts, Uganda

	Test results (RDT^a^)		Total
True disease status (Mini PCR^b^)	Negative	Positive	
Negative	30	52	82
Positive	1	17	18
Total	31	69	100

Observed agreement = 47%; Expected agreement = 37.84% Kappa value = 0.1547 Accuracy = 0.48 (C I): 0379,05822)

^a^RDT= Rapid diagnostic test

^b^PCR= Polymerase chain reaction

### Sensitivity and specificity of RDT

Using mini PCR as the reference (true state), the RDT had a high sensitivity of 94.4% (95% Confidence Interval (CI): 72.7–99.9) but very low specificity of 36.6% (95% CI: 26.20–48.0) (Table 4). The area under the receiver operating characteristic (ROC) curve was 0.655 (95% CI 0.58–0.731). The area under the ROC is interpreted as the probability that a randomly selected diseased individual animal [D+] has a greater test value than a randomly selected non diseased individual animal [D-] (assuming the distribution of the test statistic in the D + group is higher than that in the D- group). Furthermore, the RDT had a high negative predictive value of 96.8% (95% CI: 83.3–99.9) but very low positive predictive value of only 24.6% (95% CI 15.1–36.5) (Table 4).

**Table 4:** Test characteristics of RDT^a^ compared with Mini PCR^b^ in Apac and Kiryandongo districts, Uganda

Characteristics	Estimate	95% confidence interval
Sensitivity	94.4%	72.7–99.9
Specificity	36.6%	26.2–48
Receiver Operating Characteristics (ROC) area	0.655	0.58–0.731
Positive predictive value	24.6%	15.1–36.5
Negative predictive value	96.8%	83.3–99.9

^a^RDT= Rapid diagnostic test

^b^PCR= Polymerase chain reaction

## Discussion

The present study was designed to compare performance of rapid diagnostic test and PCR based assay for the detection of trypanosome infection in two cattle corridor districts of Uganda. The higher percentage of positive test results observed in Kiryandongo district compared to Apac district may be due to the fact that Kiryandongo neighbors a national park which is a high-risk area for Trypanosomosis transmission from wild animals to cattle. The national park vegetation provides a good breeding ground for tsetse flies and wild animals act as reservoir hosts for livestock infective trypanosomes. Similar findings have been reported by other studies conducted near national parks [3, 26, 27]. By contrast, Apac district is not near a national park and had lower disease risk than Kiryandongo, a finding that is consistent with those reported by a study that found low prevalence of Bovine trypanosomosis (4.3%) in Lira [28], a district that neighbors Apac and has similar ecological and farming practices.

The higher percentage of positive test results using the rapid diagnostic tests than PCR may be explained by the fact that the rapid diagnostic test detects antibodies, while PCR detects DNA of the trypanosomes. Thus, the PCR test detects current infections while the RDT detects both current and past infections [22]. This limits the use of antibody detection methods in treatment decisions compared to antigen-based methods. Antigen detection methods are, therefore, more relevant in supporting field veterinarians in making treatment decisions. However, antibody methods are useful tools for epidemiological surveillance. Trypanosome spp antibodies are usually detectable after 2–3 weeks after infection [29].

The development of a rapid diagnostic test for trypanosome infection (*T. vivax* and *T. congolense*) based on targeting more specific antigens is relatively recent. The antigen capture ELISA test for *T.vivax* and *T.congolense* using TvGM and TcCB1 proteins as antigens, respectively, were developed by CEVA: (Ceva Sante Animale Libourne France) but are not widely available [30, 31]. Unfortunately, the cost of the test is prohibitive and its availability is very limited in Uganda. With this in mind, an affordable rapid diagnostic test for animal trypanosomosis would be an alternative that would be practical under field conditions in these low resource settings. Currently, there are few points of care (POC) tests that fully meet the World Health Organization and World Organization for Animal Health ideal rapid test, ASSURED criteria: (A = Affordable; S = Sensitive; S = Specific; U = User friendly [Simple to perform in a few steps with minimum training]; R = Robust and rapid [can be stored at room temperature and results available in < 30 min]; E = Equipment free or minimal equipment that can be solar powered; and D = Deliverable to those who need them). PCR is very reliable in diagnosis of natural animal infections caused by the main trypanosoma species and subspecies [32] with sensitivity and specificity of 65–88% and 99–100%, respectively [12].

The higher prevalence of *T. vivax* compared to other species of trypanosomes may be attributed to the abundance, in this area, of the tsetse fly species (*Glossina fuscipes*) responsible for the transmission of *T.vivax* relative to other species [2, 33]. The highly significant (*p* < 0.001) Mc-Nemar’s test is strong evidence that the proportion of positive samples detected by the two tests are different which is an indication of potential bias of the rapid test. Moreover, the kappa value (k = 0.1547) indicated only slight agreement between the rapid and PCR tests. These results are consistent with those of a study by Fidelis et al. (2019) [34] who compared conventional and molecular techniques *for T. vivax* diagnosis and found the following detection rates: molecular technique (61%), parasitological method (Hematocrit centrifugation technique) (44.4%), serological methods (IFAT: 94.4%) and (ELISA: 90.7%). A related study that compared novel quantitative PCR (qPCR) and conventional PCR reported overall prevalence with qPCR assay of 11.14% compared to 91.3% with conventional PCR of Animal African Trypanosomosis in cattle [35].

Using mini PCR as a reference, RDT had better sensitivity (94.4%; 95% CI: 72.7–99.9) than specificity (36.6%; 95% CI: 26.2–48). Sensitivity and specificity are inversely related as sensitivity increases, specificity reduces and vice versa [36, 37]. These results differ from those of a study by Ngasala et al. (2019) [38] which compared PCR and Malaria RDT (mRDT) and found sensitivity of 75.9% (95% CI 62.8–86.1) and specificity of 95.7% (95% CI 92.5–97.9). Currently, PCR is widely used for the detection of trypanosomes and has proven to have a fair sensitivity (65–88%) and quite high specificity (99–100%) in laboratory settings [12]. PCR methods for identification of trypanosomes are more accurate, have high throughput and have led to better understanding of genetic diversity of trypanosome species [39]. However, the use of portable mobile PCR machines, like the one assessed in this study, have not been implemented in both districts probably due to lack of awareness of its availability, cost, or lack of trained personnel to operate the equipment. The receiver operating characteristic (ROC) curve area (0.655, CI 0.58–0.731) showed that RDT has a discriminatory capacity to diagnose animals with and without disease [40, 41]. The negative predictive value (NPV) was about four times higher than the positive predictive value (PPV). Both negative and positive predictive values depend on the pre-test probability (i.e. probability of the presence of the disease before a diagnostic test) which is determined by baseline risk factors such as disease prevalence. PPV increases with increased disease prevalence and NPV increases with decreased disease prevalence [42].

### Strengths and weaknesses

To our knowledge, this is the 1st study that has evaluated the use of rapid diagnostic tests and miniPCR for detection of trypanosomosis in Uganda. The findings of this study provide evidence that veterinarians could use these tools under field conditions to improve treatment decisions and surveillance. However, the study is not without limitations. We acknowledge that the significant McNemar’s test results indicate substantial disagreement between the predicted and actual classifications, suggesting that the Kappa statistics may not be appropriate in this context [25]. The cross-sectional study design only provides a snapshot in time. Moreover, the antibody-based methods for detection of Trypanosomatidae have low specificity as a result of cross reactions between *T. vivax*, *Trypanozoon* and *T.Congolense sesu lato* (s.l) but a higher sensitivity for other genera such like *Anaplasma*, *Babesia* and *Theileria* [13]. In another study by Magona et al. (2002) [43] that compared antibody detection ELISA technique for *T. congolense* or *T.vivax*, the *T.congolense* assay had a sensitivity and specificity of 63.7% and 57.5% while the *T. vivax* assay had a sensitivity and specificity of 82.5% and 88.7%, respectively. Despite these limitations, the use of these tests in these communities would still be beneficial considering field veterinarians in the two study districts don’t have access to any diagnostic tests and must rely entirely on clinical signs to make treatment decisions. The small sample size of 100 animals is another limitation of the study.

## Conclusions

This study found high sensitivity but low specificity of RDT using mini PCR as a reference. Considering the limited diagnostic services available to field veterinarians in these low resource and rural settings, availability of these diagnostic tests in these conditions would make a big difference in informing diagnostic and treatment decisions as well as surveillance. However, more work will need to continue to be done to better develop these rapid diagnostic tools to improve their accuracy and performance under field conditions. Although the current RDT or point of care (POC) test has some limitations, the work so far done provides a promising future of animal trypanosomosis field diagnostics in low resources settings.

## Electronic Supplementary Material

Below is the link to the electronic supplementary material.

Supplementary Material 1

## Data Availability

The dataset(s) supporting the conclusions of this article is (are) available from the corresponding author on reasonable request.
