# Supplementary material for: Comparative evaluation of rapid diagnostic test and PCR-based diagnostic assay for identification of trypanosomes in cattle of Apac and Kiryandongo districts, Uganda: A cross sectional study
Source: BMC Vet Res. 2024 Dec 19;20:570. doi: 10.1186/s12917-024-04436-7 (PMC11658059; doi:10.1186/s12917-024-04436-7)
Supplement: Supplementary file 1 — Supplementary Material 1 [file 12917_2024_4436_MOESM1_ESM.docx]

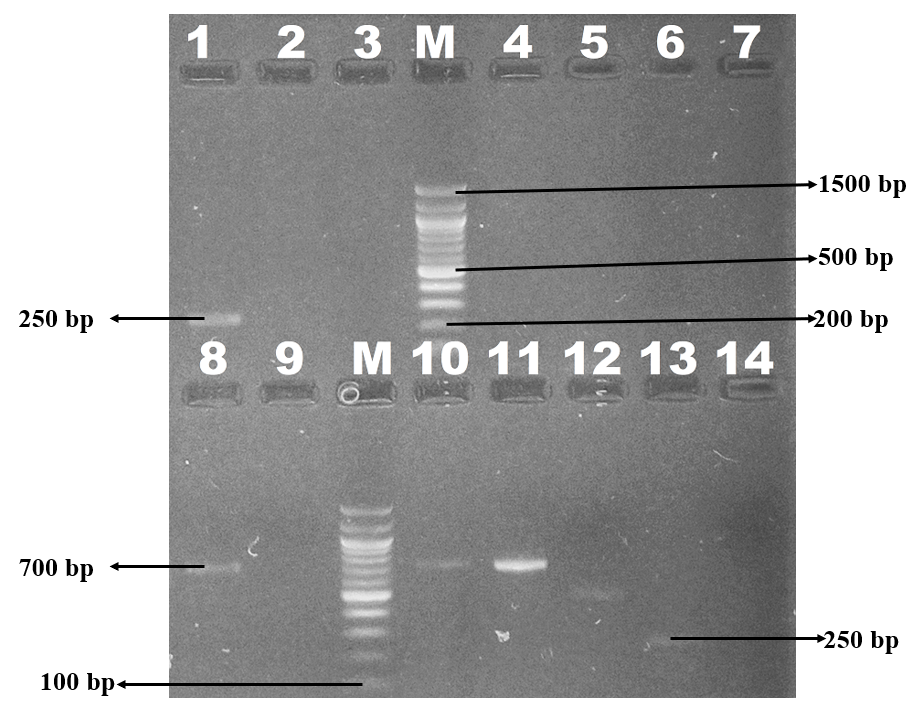


**Figure 2:** Representative gel image of the test results amplified on the miniPCR^®^ after electrophoresis on the blueGel™ Integrated electrophoresis system. Image was captured on a synegene UV transilluminator to enhance quality for publication purposes. . *Lanes*: *1* 250 *bp (T. vivax)*, *M*: 100bp DNA ladder, 4: Negative control, 8*:* 700 bp *(T. Congolese spp.)*, 12: 480 bp *(T. Brucei sl)*
